# Unbiased Proteomic Approach Identifies Unique and Coincidental Plasma Biomarkers in Repetitive mTBI and AD Pathogenesis

**DOI:** 10.3389/fnagi.2018.00405

**Published:** 2018-12-18

**Authors:** Joseph O. Ojo, Gogce Crynen, Jon M. Reed, Rosa Ajoy, Prashanthi Vallabhaneni, Moustafa Algamal, Paige Leary, Naomi G. Rafi, Benoit Mouzon, Michael Mullan, Fiona Crawford

**Affiliations:** ^1^Experimental Neuropathology and Proteomic Laboratory, Roskamp Institute, Sarasota, FL, United States; ^2^James A. Haley Veterans’ Hospital, Tampa, FL, United States; ^3^Life, Health and Chemical Sciences, The Open University, Milton Keynes, United Kingdom; ^4^Boehringer Ingelheim Pharmaceuticals, Inc., Ridgefield, CT, United States

**Keywords:** mild-TBI, Alzheimer’s disease, hTau, PS1/APP, plasma biomarker, proteomics

## Abstract

The relationship between repetitive mild traumatic brain injury (r-mTBI) and Alzheimer’s disease (AD) is well-recognized. However, the precise nature of how r-mTBI leads to or precipitates AD pathogenesis is currently not understood. Plasma biomarkers potentially provide non-invasive tools for detecting neurological changes in the brain, and can reveal overlaps between long-term consequences of r-mTBI and AD. In this study we address this by generating time-dependent molecular profiles of response to r-mTBI and AD pathogenesis in mouse models using unbiased proteomic analyses. To model AD, we used the well-validated hTau and PSAPP(APP/PS1) mouse models that develop age-related tau and amyloid pathological features, respectively, and our well-established model of r-mTBI in C57BL/6 mice. Plasma were collected at different ages (3, 9, and 15 months-old for hTau and PSAPP mice), encompassing pre-, peri- and post-“onset” of the cognitive and neuropathological phenotypes, or at different timepoints after r-mTBI (24 h, 3, 6, 9, and 12 months post-injury). Liquid chromatography/mass spectrometry (LC-MS) approaches coupled with Tandem Mass Tag labeling technology were applied to develop molecular profiles of protein species that were significantly differentially expressed as a consequence of mTBI or AD. Mixed model ANOVA after Benjamini–Hochberg correction, and a stringent cut-off identified 31 proteins significantly changing in r-mTBI groups over time and, when compared with changes over time in sham mice, 13 of these were unique to the injured mice. The canonical pathways predicted to be modulated by these changes were LXR/RXR activation, production of nitric oxide and reactive oxygen species and complement systems. We identified 18 proteins significantly changing in PSAPP mice and 19 proteins in hTau mice compared to their wild-type littermates with aging. Six proteins were found to be significantly regulated in all three models, i.e., r-mTBI, hTau, and PSAPP mice compared to their controls. The top canonical pathways coincidently changing in all three models were LXR/RXR activation, and production of nitric oxide and reactive oxygen species. This work suggests potential biomarkers for TBI and AD pathogenesis and for the overlap between these two, and warrant targeted investigation in human populations. Data are available via ProteomeXchange with identifier PXD010664.

## Introduction

Traumatic brain injury (TBI) is one of the major causes of death and disability, and is also one of the largest risk factors for neurodegenerative diseases such as Alzheimer’s disease (AD). In particular, a history of repetitive mTBI (r-mTBI) or concussive injuries, caused by contact sports or accidental injury, has been shown to precipitate clinicopathological profiles of patients many years after injury, and exacerbate neuropathological lesions observed in autopsy AD brains (Gedye et al., 1989; Mortimer et al., 1991; Fleminger et al., 2003; McKee et al., 2009, 2013; Omalu et al., 2011). While many clinical studies have evaluated the epidemiological link between TBI and AD, there is still a considerable lack of understanding concerning the molecular underpinnings between mTBI and AD. Thus, there is a paramount need to explore this inter-relationship since the ability to identify the molecular and cellular indicators of mTBI could aid risk assessment for AD, prognosis, and evaluation of treatment before a subject’s condition is exacerbated.

The diagnosis of mTBI can be very challenging for clinicians, typically because mTBI remains undetected by structural neuroimaging techniques. Blood based biomarkers are potential tools for detecting neurological changes in the brain in a non-invasive and easily accessible manner. Blood biomarkers are being successfully used in some cardiovascular, metabolic and neurodegenerative conditions as prognostic or diagnostic markers (Vasan, 2006; Yong, 2014; Lleó et al., 2015; Jeromin and Bowser, 2017). A large number of prospective blood based biomarker studies have been conducted in AD, yielding some candidate molecules that correlate with functional outcome (Thambisetty et al., 2010a,b, 2011; Kiddle et al., 2012; Velayudhan et al., 2012; Burnham et al., 2014; Ashton et al., 2015; Baird et al., 2015). However, none have been accepted as a definitive blood biomarker of AD. From the few mTBI studies which have investigated blood based biomarkers, a significant role was identified for astrocyte, axonal and proteosomal proteins in the blood at relatively acute time points post-injury (DeKosky et al., 2013; Shen et al., 2014; Kulbe and Geddes, 2016; Zetterberg and Blennow, 2016; Meier et al., 2017; Mondello et al., 2017; Oliver et al., 2018; Shahim et al., 2018). Some studies have also shown a significant role for AD-related tau protein species (total tau, caspase cleaved tau fragments, phosphorylated tau) in the blood following mTBI in athletes and military personnel (Di Battista et al., 2013; Neselius et al., 2013b; Shahim et al., 2014, 2016; Olivera et al., 2015; Rubenstein et al., 2015; Gill et al., 2018). However, most of these human studies lack adequate controls, and chronic (or longitudinal) time points that are more relevant to the current at risk patients with a history of mTBI exposures, some have also been based on acute studies and also retrospective design reports, which have inherent limitations, such as selection bias, substantial heterogeneity in patient populations, variance in type/nature of injury and post-injury intervals. These studies have also not been well-validated and reproducible by independent laboratories in different prospective cohorts of a variety of TBI patients.

Advances in the field have been observed with the FDA authorizing marketing of the first blood test for serum glial fibrillary acid protein (GFAP) and ubiquitin c-terminal hydrolase-L1 (UCHL1) to aid in the acute evaluation of concussion in adults (Okonkwo et al., 2013; Papa et al., 2016; Welch et al., 2017). However, the utility of these markers as diagnostic tools for mTBI, and their specificity remains in question as others have reported negative results (Diaz-Arrastia et al., 2014; Tenovuo et al., 2016; Mondello et al., 2017). A specific and sensitive chronic-related biomarker for asymptomatic individuals at risk for developing neurodegenerative diseases many years after exposure to a history of multiple mTBI’s also still remains lacking. Identification of such putative blood based biomarkers will be helpful for diagnosis and management of mTBI patients beyond the initial exposure to injuries. Additionally, given this poorly established relationship between repetitive mTBI and AD blood based biomarkers, there is an urgent need to identify candidate markers that would aid in early warnings and preventative therapies for r-mTBI patients if at risk for AD.

Animal models are an important early platform for conducting such studies because they can be conducted in a controlled manner without the influence of confounding variables observed in human studies. They thus facilitate more global “omic” style analyses which can be used to identify specific potential biomarkers which can then be targeted for investigation in human samples. Preclinical models also afford the design of prospective analyses of relevant biomaterials required for molecular studies at extended time points across the lifespan. Although biomarker studies have been conducted in a variety of preclinical models of TBI (Gyorgy et al., 2011; Ahmed et al., 2012; Rostami et al., 2012; Chase, 2014; Sharma et al., 2017), there remains a scarcity in longitudinal studies for monitoring chronic changes in blood biomarkers after repetitive mTBI.

To address this problem, we utilized our well established animal model of repetitive mTBI to explore putative biomarkers in the blood after injury. This model has been very well characterized up to 2-years post-injury, and shows pathological and behavioral changes comparable to those observed in human mTBI patients, typified by deficits in spatial learning, white matter damage, corpus callosum thinning, and glial activation (Mouzon et al., 2012, 2014, 2018b; Ojo et al., 2013, 2015). For AD models we used the hTau transgenic model of age-related tauopathy (mice expressing all six isoforms of human tau, on a null murine tau background) and the PSAPP mouse model of amyloid pathologies [carrying the PS1(M146L), and APP(K670N, M671L) mutations], in which emerging neurological and histopathological features have been well characterized (Holcomb et al., 1998; Duff et al., 2000; Arendash et al., 2001; Gordon et al., 2002; Andorfer et al., 2003, 2005; Sadowski et al., 2004; Trinchese et al., 2004; Polydoro et al., 2009). We have chosen to explore timepoints encompassing pre-, peri- and post-“onset” of the cognitive and neuropathological phenotypes (i.e., ages 3, 9, and 15 months in the hTau and PSAPP mice) and timepoints post-injury of 24 h, 3, 6, 9, and 12 months in the r-mTBI mice in order to capture anticipated responses overlapping with AD pathogenic changes. We used a liquid chromatography and mass spectrometry (LC/MS) proteomic based approach to interrogate plasma samples from these mouse models. An unbiased proteomics approach represents a powerful tool to bring to bear in molecular characterizations of neurodegenerative pathways for therapeutic target discovery and biomarker identification. We have successfully employed this approach to identify biomarkers/molecular targets in mouse models of other neurodegenerative diseases (Crawford et al., 2012; Zakirova et al., 2017). In this study we provide a comprehensive timeline of unique and converging molecular changes in the plasma, after repetitive mTBI, and in the AD mouse models. This work lays the groundwork for future validation and translational studies in humans, to confirm the role of these identified proteins and implicated molecular pathways as non-invasive systemic/plasma biomarkers of repetitive mTBI sequelae and possible risk for AD pathogenesis.

## Materials and Methods

### Animals

**FIGURE 1:**
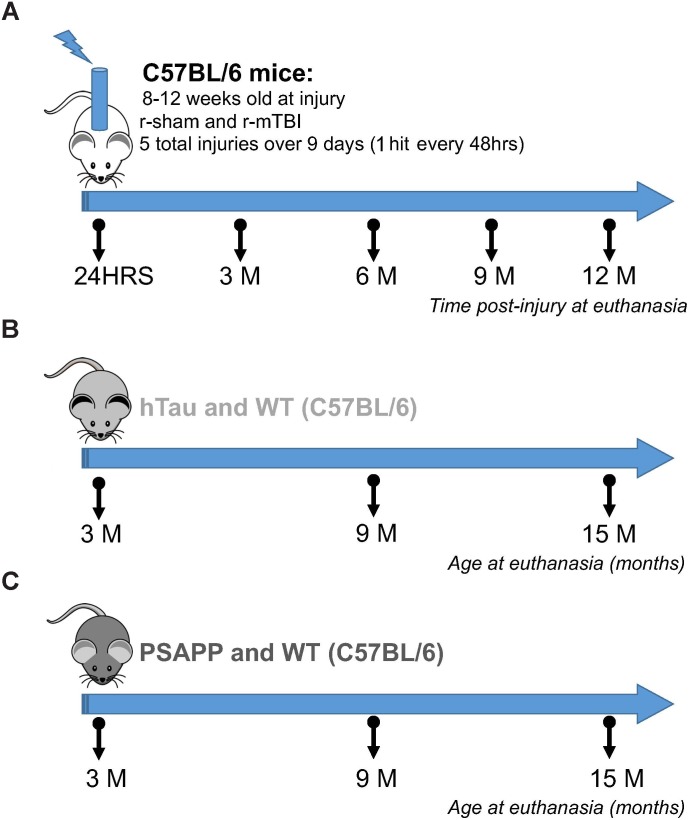
Experimental design and timeline of repetitive mTBI and AD mouse models. Six different groups of mice were used in this study. The mTBI group consisted of C57BL/6 mice exposed to sham and repetitive mTBI injury at 8–12 weeks of age, and euthanized at 24 h, 3, 6, 9, and 12 months post-injury for proteomic analyses **(A)**. The tauopathy group consisted of hTau and background strain C57BL/6 mice euthanized at 3, 9, and 15 months of age **(B)**. The amyloidogenesis group consisted of PSAPP (PS1/APP) and inbred background strain C57BL/6 mice euthanized at 3, 9, and 15 months of age **(C)**. *N* = 4 per group at each timepoint and age.

Wild-type C57BL/6 mice and hTau transgenic mice expressing human tau on a C57BL/6 and null murine tau background (generated as previously described by Andorfer et al., 2003) were purchased from Jackson Laboratories, Bar Harbor, ME. PSAPP mice expressing PS1 and APP AD-causing mutations [PS1(M146L), APP(K670N,M671L)] were bred in our vivarium facility on a C57BL/6 background, wild-type littermate controls were used as controls for PSAPP mice (see Figure 1).

hTau and PSAPP mice and their C57BL/6 littermate controls were allowed to age until euthanasia at 3, 9, and 15 months of age. C57BL/6 mice used for the r-mTBI study were 12 weeks old at the time of injury. Animals were housed in standard cages under a 12-h light/12-h dark schedule at ambient temperature controlled between 22 and 23°C under specific pathogen free conditions. Animals were given food and water *ad libitum* and maintained under veterinary supervision throughout the study. There was no evidence of disease among the colony. Male mice were randomly assigned to experimental groups with a sample size of 4 per group. All mice were male to avoid any confounding effects of gender and to limit the numbers of mice required. Experiments were performed in accordance with the Office of Laboratory Animal Welfare and National Institutes of Health guidelines under a protocol approved by the Roskamp Institute Institutional Animal Care and Use Committee (IACUC – R054). All analyses were carried out blind to study group assignment.

### Experimental mTBI

The experimental TBI methods were performed as previously described (Mouzon et al., 2012). Briefly, mice were anesthetized with 1.5 L per minute of oxygen and 3% isoflurane for 3 min. After shaving of the injury site, mice were transferred into a stereotaxic frame (Just For Mice Stereotaxic, Stoelting, Wood Dale, IL, United States) mounted with an electromagnetic controlled impact device (Impact One Stereotaxic Motorized Impactor, Richmond, IL, United States). Heads were positioned and fixed in the device, which prevented lateral movements as the impact was delivered. All mice were placed on a heating pad to maintain their body temperature at 37°C. A 5-mm blunt metal impactor tip attached to the electromagnetic motorized device was zeroed on the scalp and positioned above the midsagittal suture before each impact using the NeuroLab controller. On satisfactory positioning, the tip was retracted and the depth was adjusted to the desired level. The scalp was gently stretched by hand to restrict lateralization of the impact and to prevent the rod from delivering an inadequate trauma load at an irregular angle. Injury parameters were 5 m per second strike velocity, 1.0 mm strike depth, 200 ms dwell time, and a force of 72N. This sublethal impact does not cause direct tissue damage to the injury site, and there is no development of skull fracture or subdural hemorrhage, even after repetitive injuries. Mice in the r-TBI group received 5 hits over a 9-day period with an inter-injury interval of 48 h. Repetitive sham control mice received anesthesias of the same frequency and duration (∼3 min per session) as their r-TBI counterparts. After each impact was delivered, the mice were allowed to recover on a heating pad set at 37°C to prevent hypothermia. On becoming ambulatory, mice were returned to their cages and carefully monitored for any abnormalities.

### Plasma Protein Fractionation

A novel fractionation approach was utilized that enables albumin and hemoglobin depletion from precipitated plasma or serum samples. While approach does not achieve the same level of abundant protein depletion as can be obtained via immunoaffinity approaches, it was chosen over established solvent-based or chromatographic depletion of abundant plasma proteins. A key attribute of this fractionation protocol is that it enables downstream lipidomic and/or metabolomic sample preparation from the same sample aliquot, as there is no addition of non-volatile salts to the crude sample at any step – a critical feature when dealing with sample-limited situations such wherein poly-omic comparisons of mouse plasma are planned.

Briefly, 50 μl plasma samples were precipitated by addition of 7 volumes of methanol and centrifuged at 21,000 × *g* RCF at room temperature for 1 min, and the supernatant fraction saved. 200 μl of hexane was added to the pellet, and the pellet disrupted by bath sonication for 1 min, followed by centrifugation to pellet the insoluble material. This process was repeated, though with the addition of 2:1 isopropanol : hexane in lieu of 100% hexane. [note: These solvent supernatants were pooled, saved, and stored at -80°C for future lipidomic and metabolomic analysis by GC- and LC-MS/MS approaches]. The resultant pellet was then sonicated in the presence of 100 μl of 58% v/v hexafluroisopropanol (HFIPA) in water, and centrifuged at 20,000 × *g* RCF for 1 min. The supernatant (containing an albumin- and Hb-depleted plasma protein fraction was transferred to a new tube, and the fraction precipitated with the addition of four volumes of chilled acetone to remove the HFIPA. This pellet was re-suspended in 10X reduction/alkylation/denaturation buffer [20 mM TEAB, 1 mM TCEP, 2.5 mM C-AM, and 5% w/v sodium deoxycholate (SDC)] by bath sonication, and incubated at 37°C in darkness for 30 min to allow for simultaneous reduction and alkylation. The samples were then diluted 10-fold with addition of 20 mM TEAB, and a 20 μl process aliquot taken for protein quantification using the BCA assay, and SDS-PAGE to (1) verify the concentrations obtained by BCA assay, and (2) to ensure the consistency and integrity of the protein fractions prior to tryptic digestion and TMT labeling.

### Tryptic Digestion

Sequencing grade porcine trypsin (Promega, Wisconsin) was added to 50 μg aliquots (at 1 μg/μl final protein concentration) of depleted plasma at a 1:100 enzyme-to-substrate ratio and incubated at 37°C for 16 h. 10 μl of each was transferred to a new tube following digestion and taken to dryness in a vacuum centrifuge for anhydrous TMT labeling (below).

### Tandem Mass Tag (TMT) Labeling

Briefly, we employed a multiplexed isobaric labeling strategy to allow for simultaneous identification and quantification of proteins from multiple biological samples. Six- and 10-plex TMT labeling kits (Thermo Scientific, NJ, United States) were used for analyses of protein samples from AD and TBI mouse models, respectively. This format allowed all time points from all genotypes and treatment groups to be analyzed within the same batch. All three time points (3, 9, and 15 months) in the AD mouse models (disease vs. control) were included in the six-plex TMT kit. While all five time points (24 h, 3, 6, 9, and 12 months post-injury) in the TBI mouse model (r-sham vs. r-TBI) were included in the 10 plex TMT kit. All samples and isobaric label tags were handled blind to the experimenter. Each TMT label was dissolved in 20 μl of acetonitrile solution. Ten microliters of digested protein from each sample were taken to dryness in a vacuum centrifuge. 20 μl aliquots of each label were also dried down in the speed vacuum and subsequently re-suspended in 25 mM TEAB in acetonitrile solution. Re-suspended labels were added to dried protein samples, and allowed to incubate for 1 h at room temperature, and the reactions quenched with addition of formic acid to a final concentration of 1% v/v. Labeled samples were pooled according to their respective experimental batches and subsequently taken to dryness in a vacuum centrifuge to remove acetonitrile prior to sample cleanup.

### Sample Clean Up

Residual SDC and TEAB were removed from the samples as follows. The pooled, dried TMT-labeled protein samples were re-suspended in 100 μl of 1% formic acid in water, and centrifuged at 20,000 × *g* RCF for 1 min to remove the precipitated SDC. The supernatants were collected in new tubes, into which 400 μl of ethyl acetate was added, followed by vortexing to partition the residual SDC into the organic (upper) layer. Samples were then centrifuged at 20,000 × *g* RCF for 30 s, and upper organic layers were discarded. This was repeated three times, and the final lower phase was taken to dryness in the speed vacuum. Dried samples were re-suspended in 100 μl of 0.1% formic acid. Pooled TMT-labeled samples were concentrated and de-salted using C18 reversed phase ZipTips according to manufacturer’s protocol. ZipTip eluates were re-suspended in 20 μl of 0.1% formic acid and transferred into an auto-sampler vial, and analyzed by nano-UPLC MS on a Q-Exactive Orbitrap instrument (Thermo).

### Chromatography and Mass Spectrometry Methods (LC-MS/MS)

Samples were analyzed by LC-MS/MS (Q-Exactive) as previously described (Abdullah et al., 2011; Zakirova et al., 2017). Data dependent acquisition (DDA) settings for the experiments were as follows: full-scan MS resolution = 140,000 full width at half maximum at 200 m/z, full-scan range = 380–1250 m/z, isolation width = 1.2 m/z, higher energy C-trap dissociation relative collision energy = 29, a minimum m/z setting of 100 m/z was used for all MS^2^ spectra, MS^2^ resolution = 17 500, dynamic exclusion = 180 s, and a Top 15 high/low duty cycle was used for precursor ion selection. These settings, particularly the narrow isolation window and the ultra-long gradient, were used to minimize the deleterious effects on quantitative accuracy that result from co-isolation of isobaric precursors without resorting to MS^3^-based methods.

### Data Processing and Statistical Analysis of Proteomics Data

PMi Preview software was used to survey the data files and, if necessary, to add other modifications to the search criteria. Also, Preview results were used to choose the precursor and fragment ion mass tolerances (4 ppm, 0.02 Da, respectively) as well as dynamic modifications. The following settings were used to search the data using SEQUEST and BYONIC as the search algorithms, and Uniprot mouse database (02/2016). Dynamic modifications - Oxidation/+15.995 Da (M), Methyl/+14.016 Da (E), Deamidated/+0.984 Da (N, Q), static modifications of TMT6plex/+229.163 Da (N-Terminus, K), and Carbamidomethyl +57.021 (C). Only unique peptides were considered for quantification purposes. For SEQUEST, the Percolator feature of Proteome Discoverer, and for Byonic, the target-decoy feature, were used to set a false discovery rate (FDR) of 0.01 and peptides passing this cutoff value were exported to JMP (SAS) 8.0.2 for data cleaning and statistical analysis. Proteins identified in at least half of the total number of plexes were used for the quantitative analysis. After *ln* transformation of the raw ion counts, each channel was normalized by central tendency normalization where medians were used. The ratios were formed by dividing injured or sham mice at 3, 6, 9, and 12 timepoints with 24 h time point within the respective group; likewise, 9 and 15 month old C57BL/6, hTau and PSAPP mice were divided by their 3 months old counterparts on the plex. After the median per peptide sequence from multiple fractions were calculated, mixed model ANOVA was used to test for significant proteins changing overtime within each group. The mass spectrometry proteomics plasma data have been deposited to the ProteomeXchange Consortium via the PRIDE (Vizcaíno et al., 2016) partner repository with the dataset identifier PXD010664. *Note: For access to MS data, please use reviewer account details - Username: reviewer60134@ebi.ac.uk and Password: ma0JJzM9.*

### IPA Analysis

Datasets of significantly modulated proteins were uploaded to the Ingenuity^®^ Pathway Analysis software (IPA, Ingenuity Systems^1^) in order to map the proteins onto known networks of protein interactions to ascertain the functional significance of TBI and/or AD dependent changes in protein expression in each experimental paradigm (Krämer et al., 2014). In IPA the uploaded protein lists are assigned to established molecular pathways (“Canonical pathways”) and biological functions in the knowledgebase. The Core analysis settings were; Ingenuity Knowledge base as reference set, maximum number of molecules per network was 35, maximum number of networks for analysis was 25. Only experimentally observed knowledge was used. All species, data sources, tissues/cell lines included at the time of the analysis in IPA was considered. Core analysis identified the canonical pathways that were shown to be significantly modulated in response to TBI or AD pathogenesis as a result of significant modulation of proteins represented in those pathways. The significance of the association between the data set and the canonical pathway was measured in two ways: (1) For each canonical pathway a ratio of the number of molecules from the data set that map to that pathway divided by the total number of molecules in that canonical pathway is displayed. (2) Fisher’s exact test was used to calculate a *p*-value determining the probability that the association between the proteins in the dataset and the canonical pathway is explained by chance alone. *P*-values lower than 0.05 were considered significant.

### Enzyme-Linked Immunosorbent Assay (ELISA)

Enzyme-linked immunosorbent assay were conducted to validate proteomic results, procedures were conducted according to manufacturer guidelines.

## Results

### Temporal Changes in Unique and Common Proteins in the Plasma of Repetitive mTBI and Sham Mice Across Multiple Post-injury Time Points

**FIGURE 2:**
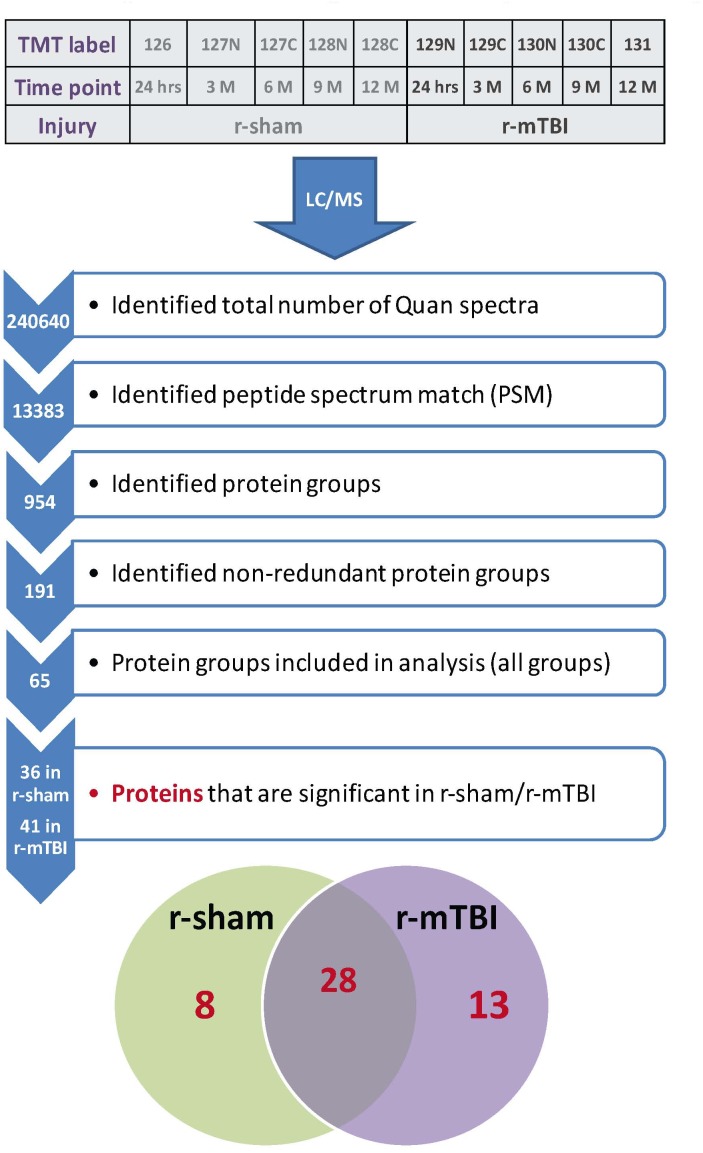
Summary of tandem mass tag (TMT) labeling, liquid chromatography/mass spectrometry (LC/MS) and proteomic analyses of plasma samples from repetitive mTBI and sham injured mice. Work flow showing randomization of samples for TMT labeling, pooling of samples for multiplexing with 10-plex TMT isotopic mass tag labels, identified total number of quantra spectra, peptide spectrum match, protein groups analyzed in all plexes, and venn diagram showing significantly regulated proteins changing across five multiple post-injury timepoints in plasma samples from sham and injured mice. Eight unique proteins were changing in sham mice with age, 13 unique proteins were changing in r-mTBI mice over-time following injury, and 28 proteins were changing in both sham and injured mice with age/time-point post-injury.

A 10 plex TMT approach was used to study the proteomic profiles in repetitive injured and sham mice at 5 multiple time points post-injury (24 h, 3, 6, 9, and 12 months; see Figure 2). A total of 191 non-redundant master protein groups were observed. We interrogated 65 proteins that were identified within all biological samples in the plexes. To explore the time dependent profile of changes within each group, and to compare temporal changes in unique and common protein profiles over multiple time points between injured and sham mice, we have analyzed our data sets using a mixed model ANOVA approach. Samples were normalized to their 24 h post-injury/sham group, and statistical analyses using mixed model ANOVA identified 8 proteins significantly changing over time only in sham mice, 13 proteins significantly changing only in injured mice over time, and 28 common proteins significantly changing in both groups overtime (Figure 2). A list of all significantly modulated proteins in sham and injured mice across multiple time points post-injury is in Table 1.

**Table 1:** Significantly modulated proteins in r-sham and r-mTBI mice across multiple time points post-injury.

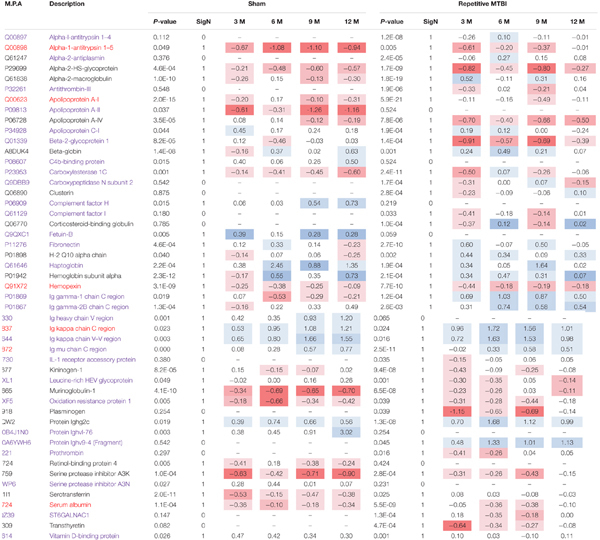

*Purple text represent lists of unique proteins significantly modulated in r-mTBI group alone. Red text represent list of proteins significantly modulated in injury and AD mouse models of tauopathy and amyloidogenesis. Values represent least squared mean generated following logarithmic transformation of calculated differences in protein expression levels after normalization with sham or injured mice from the 24 h timepoint. P-value represents FDR adjusted p-value after Benjamin–Hochberg correction and Mixed model ANOVA. SigN, statistically significant for sham or TBI groups (0 – not significant; 1 – significant); M, months; MPA, master protein accessions.*

#### To Validate Our Proteomics Data

We used an antibody-based ELISA approach to measure the levels of three selected proteins (complement factor I; Leucine-rich HEV glycoprotein, and alpha-2-macroglobulin). These proteins were selected on the basis that they were highly abundant in our samples, were significantly modulated in sham and/or injury, and easily detected by an established antibody based ELISA approach. All three proteins showed trends consistent with the TMT data; Complement I Supplementary Figure S1 (TBI increased versus sham); Leucine-rich HEV glycoprotein Supplementary Figure S2, and alpha-2-macroglobulin Supplementary Figure S3.

### Disease, Biofunctions and Canonical Pathways Modulated in Plasma Samples From Repetitive mTBI and Sham Mice Across Multiple Post-injury Time Points

**FIGURE 3:**
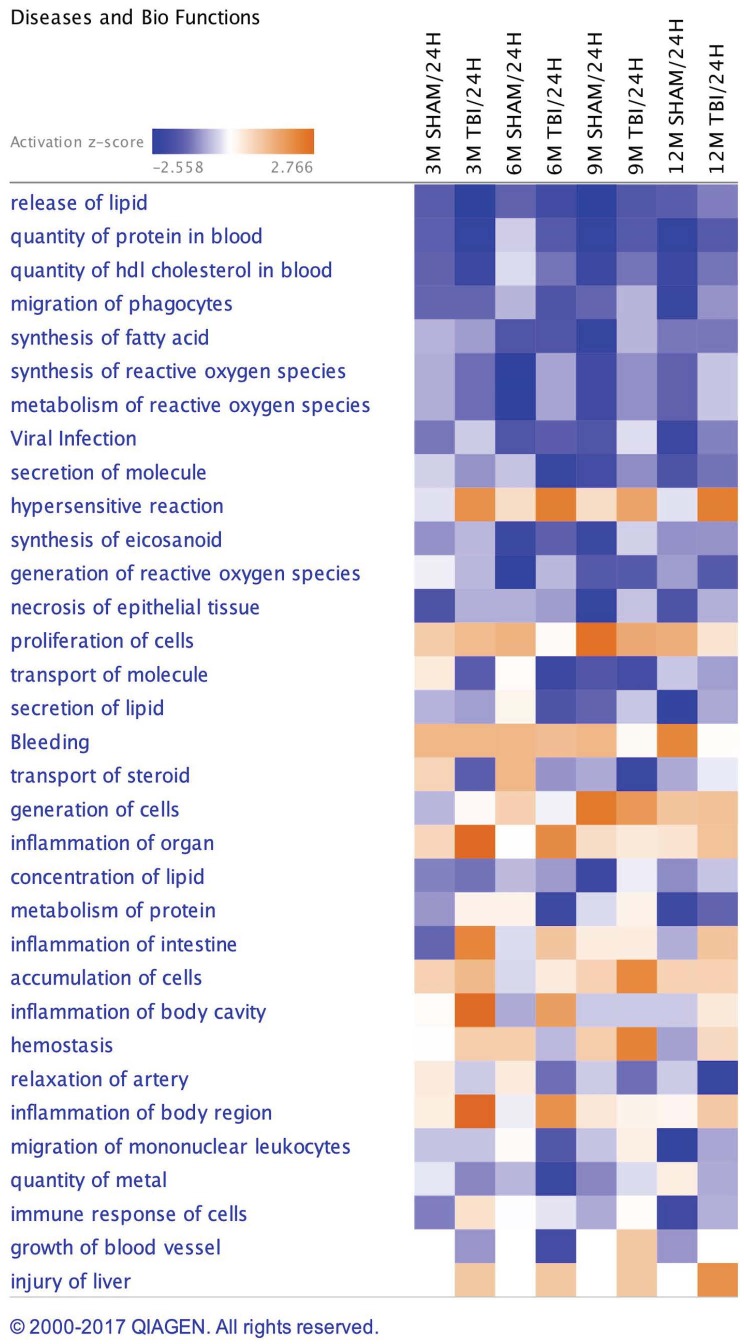
Disease and Biofunctions modulated in sham injury and repetitive mTBI plasma samples across multiple timepoints post-injury. Underlying disease pathology and biofunctions generated from the list of significantly modulated proteins in sham and injury groups across multiple timepoints using Ingenuity pathway analyses (IPA).

**FIGURE 4:**
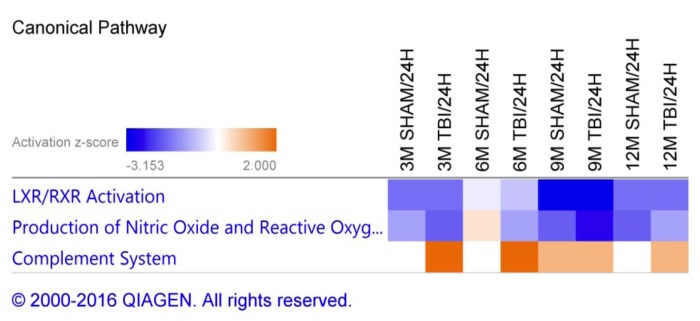
Canonical pathways modulated in sham injury and repetitive mTBI plasma samples across multiple timepoints post-injury. Identified canonical pathways generated from the list of significantly modulated proteins in sham and injury groups across multiple timepoints using IPA. Top three pathways include LXR/RXR activation, Production of Nitric oxide and Reactive oxygen species, Complement system.

A list of 33 diseases and biofunctions modulated in sham injury vs. repetitive mTBI groups across multiple time points is shown in Figure 3. Some of these diseases and biofunctions include *growth of blood vessels*, *immune response of cells*, *synthesis of eicosanoid*, *migration of phagocytes*, *synthesis and metabolism of reactive oxygen species*, *secretion of lipids*, *transport of steroids*, and *relaxation of arteries*. Ingenuity Pathway Analyses (IPA) identified the top three canonical pathways modulated in the plasma of injury vs. sham groups following analyses of significantly regulated proteins. These pathways include *LXR/RXR activation*, *Production of Nitric oxide and reactive oxygen species*, and *complement systems* (see Figure 4).

### Temporal Changes in Unique and Common Proteins in the Plasma of AD Mouse Models of Tauopathy (hTau) and Amyloidogenesis (PSAPP) at Different Ages; and Commonalities With Repetitive mTBI Model

**FIGURE 5:**
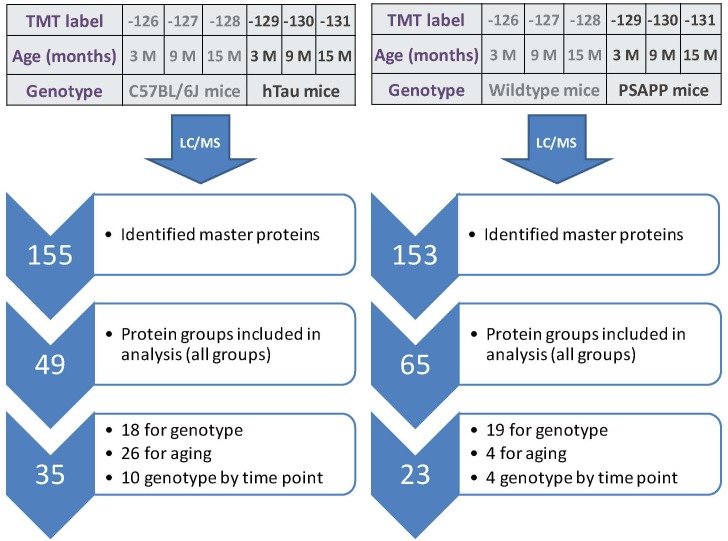
Summary of TMT labeling, LC/MS and proteomic analyses of hTau and PSAPP mouse models. Work flow showing randomization of samples for TMT labeling, pooling of samples for multiplexing with 6-plex TMT isotopic mass tag labels, identified total number of master proteins, protein groups analyzed in all plexes, and significantly regulated proteins changing across three multiple timepoint of ages in plasma samples from hTau and PSAPP mice compared to their wild type controls. In the PSAPP group, 18 proteins were changing compared to wild type mice in the combined age groups, 26 changing with age in both groups, and 10 showing an interaction between genotype and aging. In the hTau group, 19 proteins were changing compared to wild type mice in the combined age groups, 4 changing with aging in both groups, and 4 showing an interaction between genotype and aging.

A 6-plex TMT approach was used to study the proteomic profiles across hTau vs. wild-type and PSAPP vs. wild-type mice at 3 different ages (3, 9, and 15 months; see Figure 5).

A total of 153 and 155 non-redundant master proteins were identified from the PSAPP and hTau plexes, respectively; 49 proteins present in all biological samples in the hTau/WT plexes were identified and analyzed in the hTau/WT study, and 65 proteins in the PSAPP/WT study (Figure 5). We explored age-related changes in each disease genotype and compared with their wild-type controls. This was designed to compare genotype specific temporal profiles of unique and common proteins significantly altered with aging. Age-related changes were normalized to the 3-month age group for each genotype, and datasets were analyzed using mixed model ANOVA. Within the hTau group, 18 proteins were significantly altered in hTau mice compared to wild type controls in the combined age groups, 26 proteins were changing with aging and common to both hTau and WT controls, and 10 proteins showing an interaction between genotype and age (Figure 5). Within the PSAPP group, 19 proteins were significantly altered in PSAPP mice compared to their WT littermate controls in the combined age groups, four proteins were altered with aging and common to both hTau and WT controls, and four proteins showing an interaction between genotype and age (Figure 5). A list of all significantly modulated plasma proteins in the PSAPP vs. wild type groups is shown in Table 2; and likewise Table 3 shows significantly modulated plasma proteins in the hTau vs. wild type groups.

**Table 2:** Significantly modulated proteins changing with age in plasma samples from PSAPP vs. wild type littermate mice.

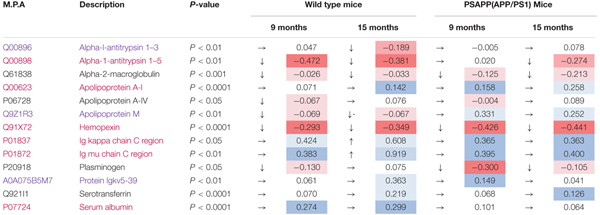

*Purple text represent lists of unique proteins significantly modulated in PSAPP group alone. Red text represent list of proteins significantly modulated in injury (repetitive-mTBI) and AD mouse models of tauopathy (hTau) and amyloidogenesis (PSAPP). Values represent least squared mean generated following logarithmic transformation of calculated differences in protein expression levels after normalization with 3 months old mice from each genotype group. P-value represents FDR adjusted p-value after Benjamin–Hochberg correction and Mixed model ANOVA. MPA, master protein accessions.*

**Table 3:** Significantly modulated proteins changing with age in plasma samples from hTau vs. C57BL/6 mice.

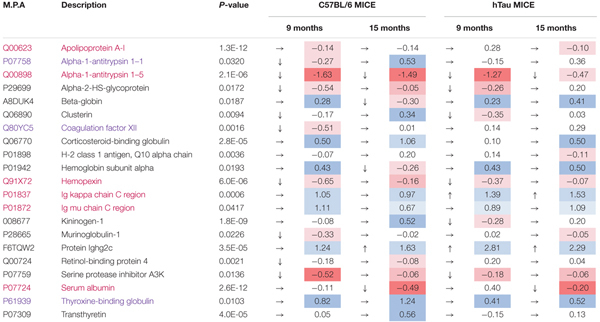

*Purple text represent lists of unique proteins significantly modulated in hTau group alone. Red text represent list of proteins significantly modulated in injury (repetitive-mTBI) and AD mouse models of tauopathy (hTau) and amyloidogenesis (PSAPP). Values represent least squared mean generated following logarithmic transformation of calculated differences in protein expression levels after normalization with 3 months old mice from each genotype group. P-value represents FDR adjusted p-value after Benjamin–Hochberg correction and Mixed model ANOVA. MPA, master protein accessions.*

We compared the significant proteomic profiles observed in all three different models to establish any unique or convergent molecular profiles between both AD models and following repetitive mTBI (see Table 4). Of the significantly regulated proteins observed to be changing with age or time post-injury in the PSAPP vs. WT littermate study (23), hTau vs. WT study (35), and the r-mTBI study (41), multiple comparisons of these proteins identified that three were unique to each of the PSAPP and hTau study, and 27 were unique to the r-mTBI study. Moreover, multiple comparisons also identified that six of these significantly modulated proteins were common to PSAPP/WT and hTau/WT group, 10 were common in the PSAPP/WT group and r-mTBI study, and eighteen were common to the hTau/WT group and r-mTBI study. Only six proteins were found to be significantly altered in all three mouse models with aging or time post-injury.

**Table 4:** List of common and unique significantly modulated proteins in plasma samples from repetitive mTBI, hTau, and PSAPP mice across all time points and ages.

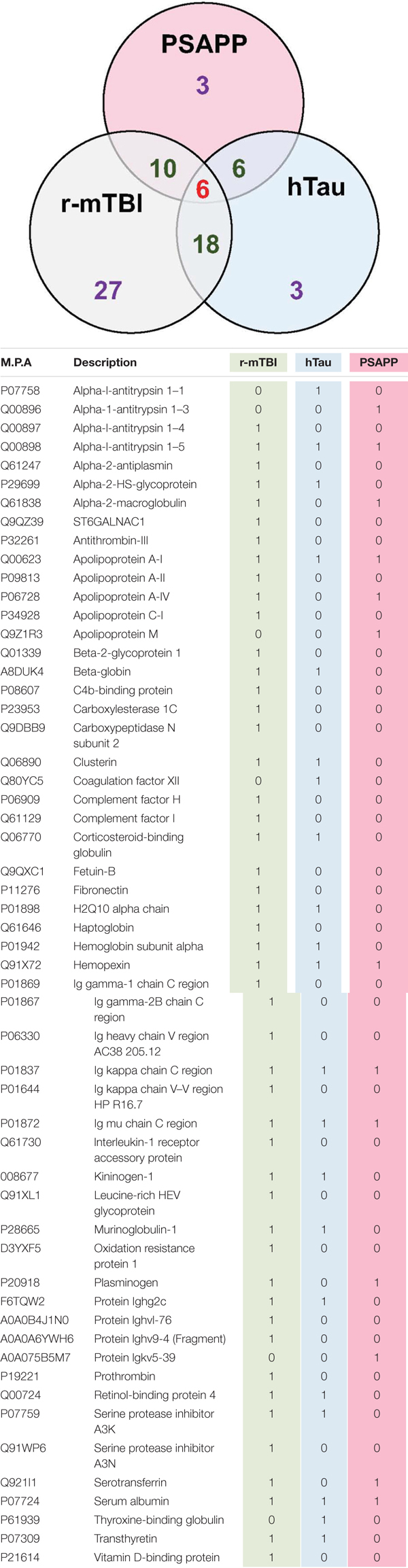

*Venn diagram and table shows number of common and unique significantly regulated proteins in all three different mouse models (0 – not significant; 1 – significant). MPA, Master protein accessions.*

**FIGURE 6:**
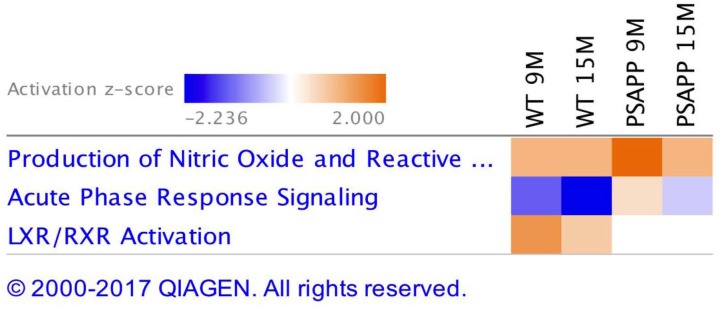
Canonical pathways modulated in PSAPP vs. wild type littermate control mice across multiple time points post-injury. Identified canonical pathways generated from the list of significantly modulated proteins in wild type and PSAPP groups across different ages using IPA. Top three pathways include Production of Nitric oxide and Reactive oxygen species, Acute phase response signaling, LXR/RXR activation.

**FIGURE 7:**
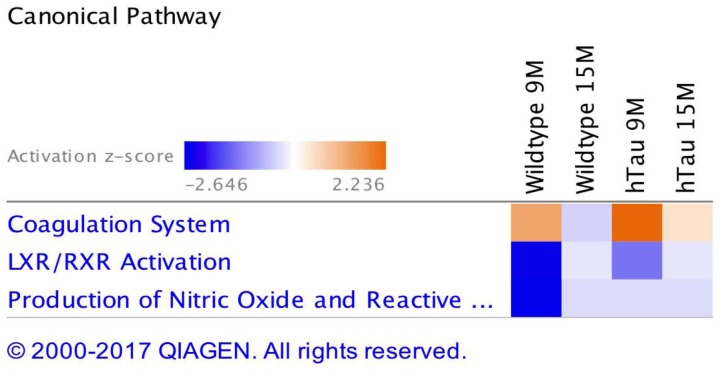
Canonical pathways modulated in hTau vs. wild type C57BL/6J mice across multiple time points post-injury. Identified canonical pathways generated from the list of significantly modulated proteins in wild type and hTau groups across different ages using IPA. Top three pathways include coagulation system, LXR/RXR activation, Production of Nitric oxide and Reactive oxygen species.

Ingenuity pathway analyses (IPA) generated three canonical pathways in each of the PSAPP and hTau studies that were significantly regulated with age compared to their WT controls, after Benjamini–Hochberg correction and fisher exact test cut-off at *P* < 0.01. In the PSAPP group these pathways were *acute phase response signaling*, *LXR/RXR activation*, and *Production of Nitric oxide and reactive oxygen species* (Figure 6). In the hTau group, the latter two canonical pathways were also in the 3 significantly modulated, along with *the coagulation system* (Figure 7). Thus, significant modulation of both *LXR/RXR activation* and *Production of nitric oxide/reactive oxygen species* was common to ALL three different mouse models.

## Discussion

This study was designed to address the lack of prospective and controlled clinical chronic studies exploring any overlap between blood-based biomarker profiles in AD pathogenesis and following repetitive mTBI. We hypothesized that a comprehensive characterization of molecular changes in the plasma of mouse models of these conditions will help to identify putative biomarkers that might have translational potential in identifying repetitive mTBI patients that are at risk for AD. We have utilized a proteomic approach to analyze blood samples from our in-house repetitive mTBI model in wild-type mice (Mouzon et al., 2012; Ojo et al., 2013; Mouzon et al., 2014, 2018a,b; Ojo et al., 2015), and the PSAPP [(PS1(M146L), APP(K670N, M671L)] and hTau [expressing all six isoforms of human tau, and lacking expression of murine tau] mouse models of AD that develop primarily amyloid and tau-based pathologies, respectively (Holcomb et al., 1998; Andorfer et al., 2003).

Our mass-spectrometry based approaches have generated a unique and detailed time-course of the molecular response to repetitive mTBI from 24 h to 12 months post-injury, and in AD models encompassing pre-, peri-, and post-“onset” of pathological phenotypes (3–15 months of age). Our data reveal unique and common mTBI-dependent and AD-progression dependent molecular level changes in the TBI and AD mouse model compared to their relevant controls.

### Unique Proteomic Changes in Blood Based Markers Following Repetitive mTBI

In the repetitive mTBI study, we identified 13 different proteins that were unique to the injury group alone and were changing over multiple time points post-injury. These proteins were mainly serine protease enzymes (such as: alpha-1-antitrypsin 1–4, alpha-2-antiplasmin, plasminogen, anti-thrombin-III, prothrombin) and metalloproteinase enzymes (such as carboxypeptidase N) which have a role in an array of biological processes, including coagulation, inflammation and biosynthesis of neuroendocrine peptides. We also identified immunoglobulins (protein ighv9-4) involved in B cell signaling, antigen binding and phagocytosis; carrier proteins involved in the transport of hormones (transthyretin and corticosterone binding globulin); molecular chaperones (such as clusterin) and inflammatory proteins (complement factor I and IL-1 receptor accessory protein). The top three canonical pathways modulated involved *LXR/RXR pathway*, *complement system* and *production of nitric oxide and reactive oxygen species*. The *LXR/RXR pathway* is involved in a host of pleiotropic effects ranging from inflammation, lipid biogenesis, energy metabolism and oxidative stress. The *complement system* is primarily involved in the innate immune response by sentinel cells. The *production of nitric oxide* and *reactive oxygen species* are byproducts of metabolism of oxygen, and their accumulation can result in oxidative stress and cellular oxidative damage. The direction of change in the levels of identified proteins in the majority of cases involved an initial trend in injured versus sham mice toward decrease at the 3-months post-injury time point compared to levels at 24 h (owing to the initial increased spike at 24 h post-injury), and thereafter a gradual increase was observed at later chronic time points (6, 9, and 12 months).

We employed an ELISA approach to validate our findings for three different proteins, which confirmed a similar trend in the groups assessed. Our previous neurobehavioral work in this injury model demonstrate deficits in spatial learning and memory processing as early as 1-week post-injury, which persists until 6 months and remains significant compared to shams at 12, 18, and 24-months post-injury time points (Mouzon et al., 2012, 2014, 2018b). Neuropathological studies also demonstrate significant axonal injury and gliosis 24 h and 3-months post-injury (Mouzon et al., 2012; Ojo et al., 2015), with the neuropathological lesions still present albeit to a lesser degree at the 6-month time point and thereafter persisting and progressively worsening between the 12–24 months post-injury time points (Mouzon et al., 2014, 2018b). These sequelae of events unique to the repetitive mTBI mice seem to echo the same pattern of changes observed in the plasma, whereby we observed an initial response consistent with ongoing brain reparative process attempting to resolve the consequences of the acute secondary injury, but failing to return to normal sham levels or phenotype, and gradually worsening into later chronic time points post-injury.

In addition to these unique r-mTBI dependent changes, we also observed that unique and distinct age-related changes in sham mice (i.e., normal and non-pathological aging profiles) were not observed in the r-mTBI group with aging, these normal age-related changes were typified by alterations in proteins involved in lipid, vitamin, and oxygen transport (apolipoprotein A-II, retinol-binding protein 4, beta globin), and regulation of serine protease activity (Fetuin-B, serine protease inhibitor A3N), implicating age-related impairment in the physiological role of these proteins in injured animals. Additionally, there were also 28 age-related proteins in our r-mTBI model that showed a common age-related pattern of change in sham mice, albeit to a different degree in most cases.

Given the very focal nature of our experimental injury to the brain, the etiology of early changes in plasma biomarkers may be directly or indirectly mediated by localized neuronal, glial or vascular injury in the brain. TBI can initially result in a transient opening of the blood brain barrier, or increased efflux of ISF/CSF drainage pathways into the blood or lymphatic vessels that can activate a series of events in the periphery resulting in changes in blood-based protein profiles. The etiology and specificity of the identified plasma changes in our mTBI model are currently unknown, and further studies will be needed to validate their utility as biomarkers and to investigate what they may contribute to our understanding of mTBI pathogenesis. Ideally the most attractive plasma biomarkers are those which can adequately track the neurological changes in the brain (irrespective of involvement in pathogenesis), and more specifically be utilized as a prognostic tool for management of mTBI patients beyond the initial exposure to injuries.

Most blood-based clinical biomarker research to date has focused on evaluating moderate to severe TBI (Kochanek et al., 2008; Svetlov et al., 2009; Dash et al., 2010; Yokobori et al., 2013), with only a few studies investigating mTBI. Because the cascade of secondary injury involves astrocytic/microglial activation, axonal degeneration, excitotoxicity, mitochondrial dysfunction, and lipid peroxidation, biomarker studies have focused primarily on identifying proteins related to these processes. For example those abundant in astroglia [S100B, Glial Fibrillary Acidic Protein (GFAP)], neurons [Neuron Specific Enolase (NSE), Ubiquitin C-Terminal Hydrolase-L1- (UCHL1)], oligodendrocytes (Myelin Basic Protein); neuronal cytoskeletal proteins (Spectrin Break Down Products, Tau, Neurofilament), inflammatory cytokines, metabolites, and oxidized lipids (Pineda et al., 2004; Kochanek et al., 2008; Svetlov et al., 2009; Dash et al., 2010; Sandler et al., 2010; Giacoppo et al., 2012; Yokobori et al., 2013; Papa et al., 2015a,b, 2016; Siman et al., 2015; Welch et al., 2016, 2017; Gill et al., 2018; Oliver et al., 2018; Shahim et al., 2018). Specifically, S100B, UCHL1, NSE, GFAP and tau have been the most studied candidates. Investigators have shown good correlation with mTBI and neurological outcome at acute time points with some of these markers (de Kruijk et al., 2001; Papa et al., 2011a,b, 2016; Berger et al., 2012; Giacoppo et al., 2012; Neselius et al., 2013b; Shahim et al., 2014, 2018; Olivera et al., 2015; Rubenstein et al., 2015; Mondello et al., 2016; Welch et al., 2016, 2017; Oliver et al., 2018). Likewise, preclinical biomarker studies in plasma samples that have focused on markers associated with the cascade of secondary injury from closed head TBI, weight drop, and blast models in rodents and pigs with a variety of frequency and injury severities have also revealed elevations in inflammatory/protease markers, astroglial, axonal, and vascular proteins (Gyorgy et al., 2011; Rostami et al., 2012; Ahmed et al., 2012; Chase, 2014; Sharma et al., 2017). Similar to the human studies, the preclinical work has largely been limited to changes at acute to subacute time points (most <3 months post-injury).

Despite the number of studies conducted to identify clinical biomarkers for TBI, very few candidates have progressed into clinical trials. The most notable markers identified by the Banyan group (GFAP and UCHL1) have shown some relative success in a few clinical studies, and has recently been authorized for marketing by the FDA as the first blood test to aid in the acute evaluation of concussion in adults (Okonkwo et al., 2013; Papa et al., 2016; Welch et al., 2017). Nonetheless, despite the success of these markers, other groups have reported negative results in clinical studies of mild CT-negative traumatic brain injury patients (Tenovuo et al., 2016), and in a multi-center TRACK-TBI observational study involving mTBI patients (Diaz-Arrastia et al., 2014; see also Mondello et al., 2017). This lack of coherent data could be attributed to the fact that some glial and neuronal proteins can be derived extracranially from other cells, while some can be released into serum during hemolysis, and others can be detected in comorbid conditions such as ischemic reperfusion injury, myocardial ischemia and non-cranial trauma (Anderson et al., 2001; Pelinka et al., 2004; Ohrt-Nissen et al., 2011). Additionally, a specific and sensitive chronic-related biomarker for asymptomatic individuals at risk for developing neurodegenerative diseases many years after exposure to a history of multiple mTBI’s also still remains lacking.

A major limitation of using CNS derived brain cell specific proteins as a TBI marker in plasma is the fact that most intracellular proteins released into the bloodstream undergo degradation and/or modification by proteases and other enzymes, and their normal half-life is unknown in most cases. Moreover, the dilution of CNS proteins into 4 L of blood containing an extraordinarily high protein concentration (60–80 g/L protein) compared to 150 ml of CSF with a protein concentration of 0.15–0.45 g/L contributes significantly to the low concentrations of CNS-derived molecules in the blood, and the difficulty in identifying consistent qualitative and quantitative changes in this complex blood proteome. This was indeed a limitation of this current study, because the majority of master protein groups identified in our plasma samples are in relatively high abundance in this biofluid, thus ultrasensitive changes to low abundant brain derived proteins in the plasma milieu may not have been adequately captured using our LC/MS approach. Nonetheless these findings do indicate that repetitive mTBI can initiate a series of persistent and unique changes in the periphery. More work will be needed to demonstrate the specificity to r-mTBI from our preclinical studies, the validity (and specificity) for human r-mTBI, whether the biomarkers are directly from brain insult or from downstream consequences of brain insult, and finally whether these insights might shed some light on the brain pathobiological sequelae.

In human studies very few unbiased proteomic approaches have been conducted in mTBI. Gao et al. (2014) evaluated serum (within 12 h post-injury) from infants that had sustained a mild abusive head injury (AHI). Using a 2D-DIGE combined with LC-MS, they identified serum amyloid A protein, an acute phase reactant and non-specific marker of inflammation that showed high specificity for AHI. This injury was, however, associated with acute extra-axial hemorrhage which is not common in most mTBI cases of this nature. Human studies have to be interpreted with caution, particularly because of inherent limitations such as the heterogeneity in population, trauma type and severity, lack of large scale prospective studies, underlying comorbidities and confounding conditions [for example, it is noteworthy to consider Plog et al. (2015) showing the impact of sleep and cisternostomy on glymphatic clearance and TBI markers]. More chronic longitudinal studies are thus needed, particularly involving very broad based unbiased approaches in a highly controlled setting to identify specific proteins that can then be investigated in a targeted fashion in the much more complex human population as we have done herein, and which underscores the value and design of our current study herein.

Longitudinal animal studies exploring unbiased biomarkers of TBI in the plasma are very sparse. We could only find our previous study on mild to severe CCI in APOE3 and APOE4 mice as an example (Crawford et al., 2012). Plasma (collected at 24 h and 1 month after injury) was analyzed using LC-MS, and 30 proteins were identified as being significantly modulated in the TBI mice over the two different time points post-injury. Bioinformatics analysis of proteins modulated after injury identified functions known to be associated with TBI such as the acute phase response, oxidative stress, and lipid metabolism, which parallel some of the changes we observed here.

### Coincidental Changes in mTBI and AD Blood Based Biomarkers

In this study we explored the areas of molecular overlap that may represent critical elements of the TBI and AD pathogenic interrelationships in the plasma. Using Ingenuity Pathway Analysis (IPA) which provides functional interpretation of observed changes, we identified key related molecules and pathways common to age-related changes in AD models and chronic post-injury changes in our mTBI model. This involved *Production of nitric oxide and reactive oxygen species*, *LXR/RXR activation* and upregulation of *inflammatory responses*. Ten and eighteen proteins were found to consistently change between mTBI vs. PSAPP models and mTBI vs. hTau models, respectively. Some of these proteins include alpha-1-antitrypsin, apolipoprotein A-1, hemopexin, Ig *k* chain C, Ig μ chain C, serum albumin, serotransferrin, plasminogen, alpha-2-macroglobulin, corticosteroid-binding globulin, clusterin and transthyretin. These significantly regulated proteins are primarily involved in (i) sequestration and precipitation of proteinaceous aggregates; (ii) mediating clearance by receptor mediated endocytic mechanisms; (iii) antigen presentation; (iv) mediating inflammation/oxidative stress mechanism; and (v) transporter proteins. The changes observed in both hTau and PSAPP models were present in older mice (aged 9–15 months) when neuropathological lesions correlate strongly with cognitive deficits. For example, PSAPP mice reportedly show normal acquisition in learning and working memory in the radial arm water maze between 6 and 9 months when neuropathology is minimal, but exhibit progressive, age-related deterioration in the performance of these cognitive tasks by 15–17 months (Holcomb et al., 1998; Arendash et al., 2001; Gordon et al., 2002). hTau mice begin to demonstrate impairment in normal object-recognition memory and spatial learning/memory assessed by the Morris Water Maze by >12 months of age (Polydoro et al., 2009). To our knowledge our work is the first comprehensive unbiased proteomic approach used to identify common blood biomarkers in a controlled longitudinal preclinical study in TBI and AD models.

In support of our findings, human studies over the last decades have implicated several plasma proteins in AD pathobiology (Baird et al., 2015), some of which we have identified in both our TBI and AD mouse study. Increased Apolipoprotein IV levels, a lipoprotein that facilitates uptake of amyloid protein has been identified in serum samples of AD (Yang et al., 2012), and codon 360 mutation in patients has also been associated with AD (Csaszar et al., 1997). Gupta et al. (2013) showed that the levels of another apolipoprotein plasma protein [ApoE] in AD revealed an obvious relationship between its levels and AD diagnosis. Lipoprotein, clusterin has been correlated with brain atrophy, and MCI, and is found in blood and brain tissue surrounding amyloid plaques (Thambisetty et al., 2010a, 2012; Jongbloed et al., 2015). Variants in clusterin are associated with the risks of AD (Harold et al., 2009; Lambert et al., 2009). Transthyretin, a carrier protein for thyroxine and retinol in plasma and cerebrospinal fluid (CSF) that sequesters proteinaceous aggregates is reduced in AD (Sousa et al., 2007; Thambisetty et al., 2010b; Velayudhan et al., 2012). Protease scavenger, alpha-2-macroglobulin has also been identified in plasma and CSF biomarker studies, and can be found in senile plaques, it has also been genetically associated with AD (Blacker et al., 1998; Kovacs, 2000; Lovestone et al., 2009; Zabel et al., 2012; Hye et al., 2006, 2014). Iron binding protein, serotransferrin which mitigates the impact of iron on precipitating amyloid aggregation has been reported in the CSF and blood of MCI and AD patients (Wildsmith et al., 2014). Other proteins reported include increased serum α-1-antichymotrypsin that participates in the inflammatory cascade of AD and enhances the formation of amyloid fibrils (Kamboh et al., 1995, 2006; Guan et al., 2012; Tyagi et al., 2013); upregulation in interleukins (IL-1α, IL-6) (Du et al., 2000; Grimaldi et al., 2000; Leung et al., 2013) and downregulation of activity-dependent neuroprotective proteins (Yang et al., 2012). The findings from the majority of these human biomarker studies seems to corroborate with our work herein, as we have demonstrated similar protein changes to these human AD plasma proteomic studies. Further studies will be needed to capitalize on our vast preclinical molecular library of omic data, to explore prospective and translatable approaches for validation and utility of putative biomarkers that may provide an insight into the link between TBI and increased AD risk.

### Limitations

A limitation of this study and other plasma proteomic studies is the efficient and comprehensive depletion of abundant plasma proteins, as it is well-known that their presence in a sample can hamper efforts to identify and quantify novel circulating biomarkers which are typically much lower in abundance. We used an approach described in the Material and Methods section – namely, the use of a 58% HFIPA solution to selectively re-suspend an essentially albumin- and hemoglobin-depleted protein fraction from a methanolic precipitate, which enables the generation of what is typically the “throwaway pellet” that could be used as part of a larger poly-mic (i.e., lipidomic and metabolomics) workflow from a single aliquot of material, which we planned for this current study. We have previously described this approach at the American Society for Mass spectrometry conference (Reed et al., 2013, 2014), and a separate manuscript describing this approach is under construction (Reed et al., *pers comm*). While we acknowledge that the protein fractionation component of our overall workflow may not perform as well as conventional immunoaffinity depletion protocols (example., Top12 or Mouse 3 spin columns from Pierce or Agilent, respectively), we have observed that it performs on par with other solvent-based depletion protocols (Reed et al., 2013, 2014), such as the modified Cohn precipitation first described by the van Eyk group (Fu et al., 2005, 2007), and subsequently modified and adapted for use in our plasma proteomics work (Crawford et al., 2012).

Technical reproducibility was key to this protocol during development and in-house validation, and we observed highly consistent sample preparations from technician to technician using pooled plasma samples. As technical variation was exceedingly minimal, we are confident in the assessment that sample-to-sample variations which are observed are primarily rooted in the sample itself. Transferrin and IgG proteins do, however, co-purify using this fractionation, as we have yet to devise additional steps to reproducibly ensure their removal. We thus acknowledge that this obviously contributes to DDA-LCMS sampling bias, and is an acknowledged limitation of the assay. In our data presentation we have listed several known abundant plasma proteins as being differentially expressed following statistical filtering. This was done to attempt to minimize bias as much as possible, and include proteins – abundant or not – into such tables as they are what the statistical analyses and the TMT data has indicated were unique to treatment groups. Thus removing them based on pre-suppositions regarding their relevance as a function of abundance, would have added an additional layer of bias, as well as compromise the intentions of our statistical analysis. To that end, we performed validations on a subset of these markers using an orthogonal bioanalytical approach (ELISA), and observed that the trends observed in the TMT analysis were recapitulated using an immunological assay.

Additionally, we also acknowledge that the models we have used may not directly represent the best platforms to explore the increased risk of repetitive mTBI contributing to the development of AD related amyloid and tau pathogenesis. This is particularly because we have used wild-type mice in our studies which do not develop tau pathogenesis nor amyloid plaques. We have examined this repetitive mTBI paradigm in our previous work at 24 h, 6, 12, 18, and 24 months post-injury, and report no tau or amyloid pathology from our comprehensive histopathological and biochemical analyses (Mouzon et al., 2012, 2014, 2018b). We recognize that more relevant humanized hTau or APP knock in mice will be required to properly address this question, as such we acknowledge that we cannot definitively describe the overlapping changes in our model as attributed to increased repetitive mTBI risk for AD pathogenesis. Previous studies have demonstrated the behavioral profiles in hTau and PSAPP mice as we have previously described (see above), and it is worth mentioning that we have also previously documented behavioral (i.e., persistent spatial learning impairment) and histopathological findings (typified by persistent axonal injury and gliosis) in our wild type mouse model from 24 h to 24 months post-injury, indicating that behavioral deficits in this model are mediated by a non-tau/amyloid mechanism. Indeed, in this same cohort we have conducted proteomics analyses of brain tissue at the same time points of analyses described in this current work, and we present distinct and overlapping proteomic response to injury in our TBI model compared with AD mouse models, indicating a distinct TBI mediated neurodegeneration (not related to tau or amyloid pathology), that also shares features with AD pathogenesis (Ojo et al., pers comm). One of the main common features observed in both TBI and AD models from our proteomic work is glial pathobiology which we are exploring in current work.

## Conclusion

We hypothesized that controlled preclinical studies in mouse models that demonstrate TBI sequelae and AD pathogenesis would identify areas of molecular overlap that may represent critical elements in the pathogenic interrelationship between TBI and AD. Our unbiased mass-spectrometry based proteomic approach has generated extensive qualitative and quantitative data sets that provide a unique and detailed time-course of the molecular response to repetitive mTBI and AD pathology, in mouse models, at a level not previously examined. Our comprehensive in depth interrogation has identified *novel longitudinal plasma biomarker profiles in our injury model. We also reveal TBI-dependent molecular overlap in our injury model which are consistent with age-dependent changes in our AD models.* The unique and convergent molecules in the TBI and AD plasma profiles represent candidates for investigation in human prospective studies as potential biomarkers that could aid risk assessment for TBI patients (such as informed decision-making regarding return to duty and deployment choices for active duty soldiers, and return to play for athletes involved in contacts sports) and possible AD prognosis, including evaluation of treatment (as theragnostic or pharmacodynamics endpoint markers) before a patient’s condition is exacerbated. Given the lack of tau and amyloid pathogenesis in our wild type TBI model, the impact of repetitive mTBI on AD risk biomarkers should be interpreted with caution due to the lack of existent AD pathology in our model. Our brain proteomic studies (Ojo et al., *pers comm*) have indicated a distinct TBI mediated neurodegeneration, which shares some overlap with AD pathogenesis in the hTau and PSAPP models, that is not primarily mediated by tau or amyloid pathogenesis in our model. We thus acknowledge that further studies will be required in humanized hTau and APP knock in mice to further explore the role of these overlapping biomarkers as a TBI induced AD risk. Additional studies will also be needed to assess the specificity and selectivity of these biomarkers in diagnosing mTBI and predicting outcome in longitudinal cohorts of mTBI patients. The biomarkers themselves, and their up- and down-stream mediators, may shed light on mTBI pathogenesis, which will first be explored in additional preclinical studies.

## Author Contributions

FC and MM conceived the project. FC, JR, and JO both directed the project. FC, JO, GC, and JR planned the experiments in the whole study. JO, FC, GC, JR, and NR were involved in the preparation of the manuscript. FC, JO, and BM participated in the establishment of the animal models. JO, RA, PV, MA, and PL performed the experiments. GC and JO participated in the analysis of the experimental data. All authors contributed to the manuscript.

## Conflict of Interest Statement

The authors declare that the research was conducted in the absence of any commercial or financial relationships that could be construed as a potential conflict of interest.

## Footnotes

1 www.ingenuity.com

## Supplementary Material

Figure S1 Validation of changes in plasma complement factor I expression levels using antibody based ELISA approach in repetitive mTBI mice at multiple time points post-injury. **(A)** Represents ratio of BCA corrected ELISA concentration values normalized to 24 h post-injury time point for repetitive mTBI only group. **(B)** Represents values from proteomic analyses using TMT labeling normalized to 24 h post-injury time point for repetitive mTBI only group.

Figure S2 Validation of changes in plasma LRG expression levels using antibody based ELISA approach in repetitive mTBI mice at multiple time points post-injury. **(A)** Represents ratio of BCA corrected ELISA concentration values normalized to 24 h post-injury time point for repetitive mTBI only group. **(B)** Represents values from proteomic analyses using TMT labeling normalized to 24 h post-injury time point for repetitive mTBI only group.

Figure S3 Validation of changes in plasma alpha-2-macroglobulin expression levels using antibody based ELISA approach in sham and repetitive mTBI mice at multiple time points post-injury. **(A)** Represents ratio of BCA corrected ELISA concentration values normalized to 24 h post-injury time point for sham and repetitive mTBI only group. **(B)** Represents values from proteomic analyses using TMT labeling normalized to 24 h post-injury time point for sham and repetitive mTBI only group.
