# Supplementary figures and images for: Unbiased Proteomic Approach Identifies Unique and Coincidental Plasma Biomarkers in Repetitive mTBI and AD Pathogenesis
Source: Front Aging Neurosci. 2018 Dec 18;10:405. doi: 10.3389/fnagi.2018.00405 (PMC6305374; doi:10.3389/fnagi.2018.00405)

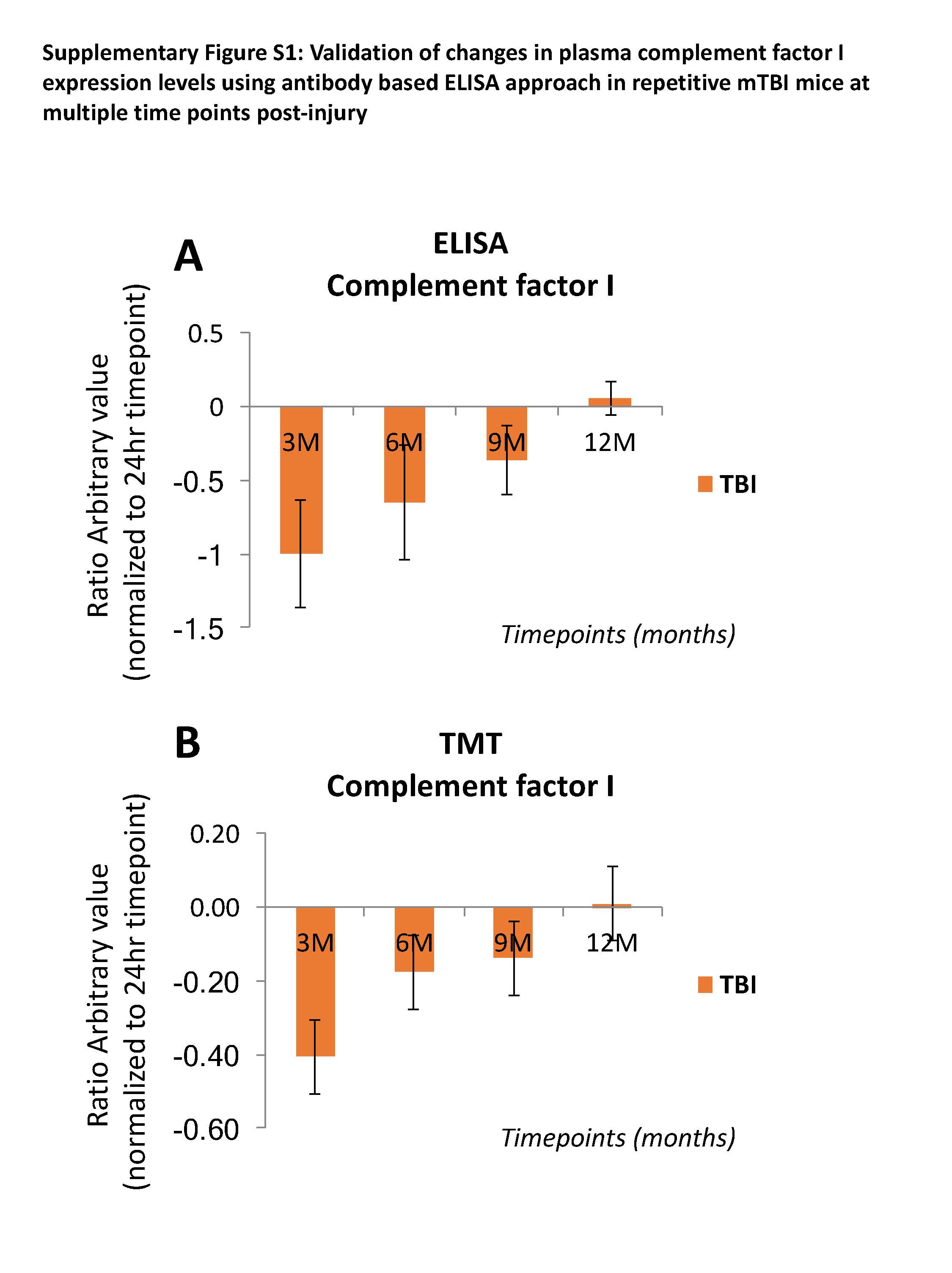

Supplement: Figure S1 — Validation of changes in plasma complement factor I expression levels using antibody based ELISA approach in repetitive mTBI mice at multiple time points post-injury. (A) Represents ratio of BCA corrected ELISA concentration values normalized to 24 h post-injury time point for repetitive mTBI only group. (B) Represents values from proteomic analyses using TMT labeling normalized to 24 h post-injury time point for repetitive mTBI only group. [file Image_1.JPEG]

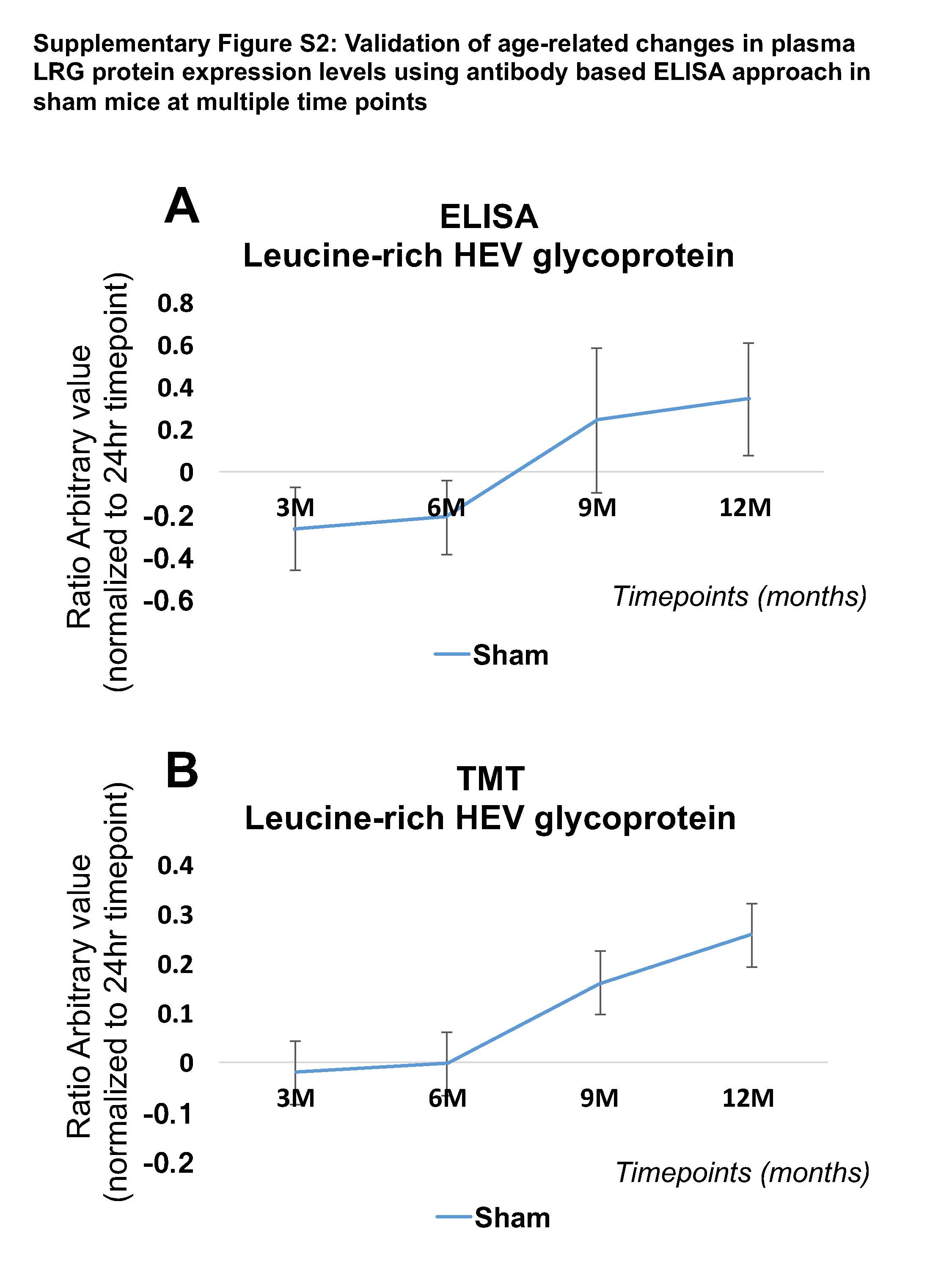

Supplement: Figure S2 — Validation of changes in plasma LRG expression levels using antibody based ELISA approach in repetitive mTBI mice at multiple time points post-injury. (A) Represents ratio of BCA corrected ELISA concentration values normalized to 24 h post-injury time point for repetitive mTBI only group. (B) Represents values from proteomic analyses using TMT labeling normalized to 24 h post-injury time point for repetitive mTBI only group. [file Image_2.JPEG]

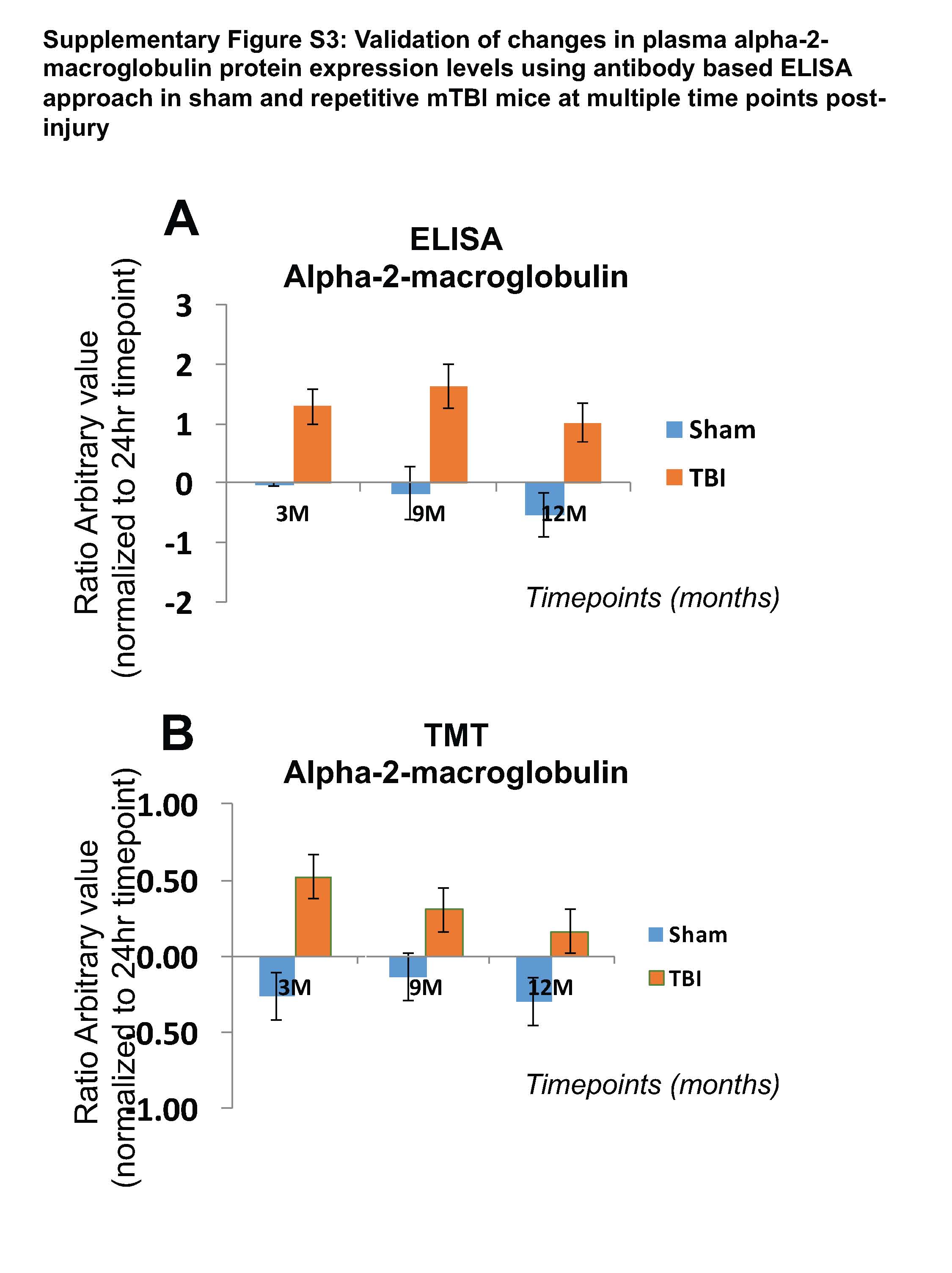

Supplement: Figure S3 — Validation of changes in plasma alpha-2-macroglobulin expression levels using antibody based ELISA approach in sham and repetitive mTBI mice at multiple time points post-injury. (A) Represents ratio of BCA corrected ELISA concentration values normalized to 24 h post-injury time point for sham and repetitive mTBI only group. (B) Represents values from proteomic analyses using TMT labeling normalized to 24 h post-injury time point for sham and repetitive mTBI only group. [file Image_3.JPEG]
